# ELECTRA-DTA: a new compound-protein binding affinity prediction model based on the contextualized sequence encoding

**DOI:** 10.1186/s13321-022-00591-x

**Published:** 2022-03-15

**Authors:** Junjie Wang, NaiFeng Wen, Chunyu Wang, Lingling Zhao, Liang Cheng

**Affiliations:** 1 Department of Medical Informatics, School of Biomedical Engineering and Informatics, Nanjing Medical University, Nanjing, People’s Republic of China; 2 School of Mechanical and Electrical Engineering, Dalian Minzu University, Dalian, People’s Republic of China; 3 Faculty of Computing, Harbin Institute of Technology, Harbin, People’s Republic of China; 4 NHC and CAMS Key Laboratory of Molecular Probe and Targeted Theranostics, Harbin Medical University, Harbin, People’s Republic of China; 5 College of Bioinformatics Science and Technology, Harbin Medical University, Harbin, People’s Republic of China

**Keywords:** Deep learning, Representative learning, Drug-target affinity prediction, ELECTRA

## Abstract

**Motivation:**

Drug-target binding affinity (DTA) reflects the strength of the drug-target interaction; therefore, predicting the DTA can considerably benefit drug discovery by narrowing the search space and pruning drug-target (DT) pairs with low binding affinity scores. Representation learning using deep neural networks has achieved promising performance compared with traditional machine learning methods; hence, extensive research efforts have been made in learning the feature representation of proteins and compounds. However, such feature representation learning relies on a large-scale labelled dataset, which is not always available.

**Results:**

We present an end-to-end deep learning framework, ELECTRA-DTA, to predict the binding affinity of drug-target pairs. This framework incorporates an unsupervised learning mechanism to train two ELECTRA-based contextual embedding models, one for protein amino acids and the other for compound SMILES string encoding. In addition, ELECTRA-DTA leverages a squeeze-and-excitation (SE) convolutional neural network block stacked over three fully connected layers to further capture the sequential and spatial features of the protein sequence and SMILES for the DTA regression task. Experimental evaluations show that ELECTRA-DTA outperforms various state-of-the-art DTA prediction models, especially with the challenging, interaction-sparse BindingDB dataset. In target selection and drug repurposing for COVID-19, ELECTRA-DTA also offers competitive performance, suggesting its potential in speeding drug discovery and generalizability for other compound- or protein-related computational tasks.

**Supplementary Information:**

The online version contains supplementary material available at 10.1186/s13321-022-00591-x.

## Introduction

Drug discovery and development are laborious, time-consuming, expensive and challenging processes. One of the most important steps in developing a new drug or repurposing existing drugs is target identification and validation. A direct brute-force search is unrealistic because of the very large number of drug-like compounds and possible drug targets. Given current advances in computational methods and techniques, especially the application of machine learning in chemical and biological research fields, computer-aided methods may be a great opportunity to shorten the drug discovery process by significantly narrowing the search space.

Apart from the prediction of binary interaction relationships between compound-protein pairs, another crucial factor in candidate screening, the prediction of the binding affinity of compound-protein pairs, also called drug-target affinity (DTA), remains a challenge in drug discovery. Laboratory experiments conducted to measure the affinity value for a large-scale drug-target pool remain time consuming and expensive. Hence, computational methods to perform binding affinity prediction have received increasing attention in recent years, and much effort has been made to accurately quantify the strength of binding for compound-protein pairs based on machine learning or deep learning. Gradient boosting machines are used in quantitative structure-activity relationship studies for regression and classification problems. SimBoost [1] employs gradient boosting machines with novel feature engineering to extract new features from drugs, targets and drug-target pairs in training datasets, and then these features are used as inputs to models to predict the binding affinity for unknown pairs. Regularized least-square (RLS) is another efficient model with various applications. The KronRLS [2] model amends RLS with the Kronecker products of drug-drug and protein-protein interactions to speed up model training for DTA prediction and has achieved promising performance. The Kronecker product part of the KronRLS is a similarity-based method in which any similarity measure could be used.

Recently, inspired by successful applications in diverse research fields, deep learning approaches have also been intensively used in bioinformatics and cheminformatics, especially in drug discovery. The first deep learning-based DTA prediction model was DeepDTA [3], which uses simplified molecular input line entry system (SMILES), a one-dimensional representation of the drug compound chemical structure, as drug features, while the protein amino acid sequences are used to represent protein features. Furthermore, the drug SMILESs are labelled as encoded integer vectors and as protein sequences. The DeepDTA model uses a CNN with three 1D convolutional layers with pooling for drug embedding to learn latent features for each drug and an identical CNN for protein embedding. Then, each pair of drug-target feature vectors is concatenated and fed into fully connected layers for training and prediction. Another novel deep learning model for DTA is DeepAffinity [4], which represents drugs with SMILESs and proteins with structural property sequences. Because of the detailed structural information and higher resolution of sequences, DeepAffinity benefits DTA regression tasks. The drug SMILESs and protein structural sequences are both encoded into embedding representations by a recurrent neural network (RNN) auto-encoder model named seq2seq [5]. The seq2seq model maps raw sequences into vectors that are learned in an unsupervised fashion to capture dependencies in sequences of SMILESs or protein residues.

The aforementioned DTA models mainly focus on developing diverse neural network architectures to learn the hierarchical feature representations on given, known CP pairs with binding affinity values in a non-handcraft manner. These existing models usually take predefined compound molecular and protein descriptors as input features, especially SMILES strings and protein residue sequences. To be fed into deep networks such as CNNs or LSTMs, the raw sequence or SMILES strings are encoded by one-hot vectors, physicochemical property-aware encoding or static embeddings. Such mechanisms deploy fixed representations, which makes the model characterize each amino acid or atom independently but do not consider the contextual information and accordingly do not highlight those that are critical to the whole protein or molecular compound. Applicable experimental structures of protein complexes are abundant, but the number of available labelled drug-target pairs remains limited. To address the issue of insufficient labelled data, unsupervised encoding methods have recently been considered for proteins or compounds. The idea behind this approach is to use protein sequences and compound SMILESs or fingerprints as a codified language for human experts with limited words and grammar. Similar to a linguist who extracts hidden knowledge from sentences of a natural language, the structure and function of chemical compounds and protein sequences can be processed deliberately to build novel solutions, such as DTA predictions, based on the level of understanding. State-of-the-art representation models for NLP, such as BERT [6], have greatly improved downstream NLP tasks due to their advantages. The recent transformer-based ELECTRA [7] model uses training data efficiently and shows better performance than BERT. ELECTRA employs a generative-discriminative model to make better use of training data; a generator model replaces some tokens in the corpus, and a co-trained discriminative model detects these replacements. Because the corpus is well used in this fashion, ELECTRA is more efficient than BERT with comparable model sizes, especially small models. This paper proposes ELECTRA-based encoders for molecular SMILESs and protein sequences separately and accordingly yields a novel DTA prediction framework incorporating an ELECTRA encoding layer, a CNN network and a regression block. The proposed framework enhances the feature representation capability as follows. First, the ELECTRA encoding layers used to represent each molecule can take advantage of existing chemical structure knowledge by pretraining on the PubChem database to extract useful chemical information, and a similar prompt occurs for the amino acid representation. Finally, the ELECTRA encoding provides the context-related representation for protein sequences and compound SMILESs, which can characterize the diversity of each atom or amino acid in a variety of sequences or strings.

In summary, our method consists of two major building blocks: one for feature representation and another for regression prediction. The first block, using two pretrained ELECTRA models, extracts local context information from drug SMILES strings and raw protein sequences separately. Then, the learned representations for both drugs and targets are passed into the second block, which uses a fully connected neural network to predict the binding affinity as a regression task. Our framework needs neither expert knowledge nor the 3D structure of the targets, so it is more convenient than existing frameworks. Additionally, the proposed framework takes advantage of the local chemical context information of atoms in drugs or amino acids in proteins, which differentiates ELECTRA-DTA from existing deep learning models. The main contributions of this paper are as follows:

1. We adopt ELECTRA, a state-of-the-art NLP model, to extract feature representations from raw sequence data.
2. We leverage a squeeze-and-excitation convolution neural network block stacked over three fully connected layers to capture the sequential and spatial features from the matrix encoded by the pretrained ELECTRA models for the DTA regression task.
3. We applied the ELECTRA-DTA model for target selection and drug repurposing for COVID-19 and again obtained competitive performance.

## Methods

In this section, we first summarize the whole model. Then, we introduce the input representation for compounds and proteins. After that, we show the training for ELECTRA and the representation tensor of compounds and proteins. Finally, we provide the details of our training and prediction model.

### Overview of the ELECTRA-DTA model

**Fig. 1:**
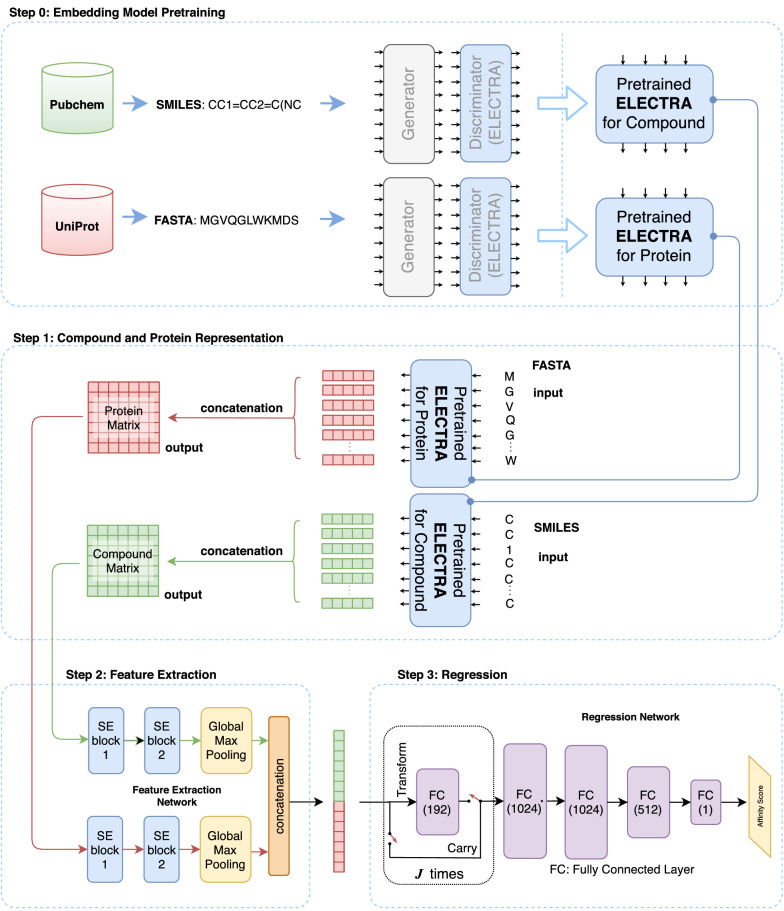
Overview of ELECTRA-DTA

Figure 1 shows the overview of ELECTRA-DTA. It takes the SMILES strings of compounds and the amino acid sequences of proteins as the input. By incorporating a pre-trained ELECTRA model, ELECTRA-DTA encodes these sequences into feature tensors as internal representations. Then, the model exploits a CNN network to learn from known drug-protein pairs in a supervised manner. Finally, it outputs the binding affinity for new pairs. The design of ELECTRA-DTA includes three main steps:

1. The training of two ELECTRA models to encode all amino acids for protein sequences and characters in SMILESs for compounds separately;
2. The encoding of the whole sequence of compounds and proteins as feature tensors;
3. Model training and predicting the binding affinity with the proposed deep neural network.

### Pretrained ELECTRA models for encoding protein sequences and compound SMILES strings

To encode the SMILESs/amino acid sequences, existing deep learning approaches such as DeepDTA and DeepCDA use label/one-hot encoding to represent each symbol in the SMILES/amino acid sequence. However, label/one-hot encoding often neglects the context of the symbol and thus cannot reveal the functionality of the symbol within the context.

#### Construction of the compound SMILES corpora

We need to train the ELECTRA model with an appropriate corpus for a specific task in advance to obtain an encoding layer for input sequences. Compound SMILES are linguistic constructs with a simple vocabulary (only atoms and chemical bond symbols) and a few grammar rules. Similar to natural language processing, compound SMILES strings are analogous to sentences, where each atom and bond symbol is a word (token). A corpus can then be naturally composed by collecting numerous compounds. For example, the SMILES string “CC(=)OC1=C” can be listed as a sentence composed of the tokens ‘C’,‘C’,‘(’,‘=’,‘)’,‘O’,‘C’,‘1’,‘=’,‘C’. The corpus we constructed contains all canonical SMILES extracted from PubChem [8–10] with a total number of 1114424.

To ensure the consistency of all SMILES from different sources, we use Open Babel [11] v3.1.0 to convert all SMILES strings in the corpora into a canonical format, which is also applied to SMILES in all datasets. Detailed information on the SMILES corpora we constructed is listed in Table 1.

**Table 1:** Statistics of the compound SMILES corpora

No. of corpus	Average length of the corpus	Minimum length of the corpus	No. of vocabulary
1114424	47	3	72

#### Construction of the protein FASTA sequence corpora

Similar to the compound SMILES, a 1-mer schema is exploited to extract words from the protein FASTA sequences and create a protein sequence corpora; in this way, each residue is a single word. We utilized the Swiss-Prot dataset from the UniProt knowledgebase [12] (UniProtKB), which included 563,552 manually annotated and reviewed protein sequences, to collect the proteins. UniProtKB/Swiss-Prot is a high-quality annotated and non-redundant protein sequence database. The detailed information on the protein sequence corpora we constructed is listed in Table 2.

**Table 2:** Statistics of protein sequence corpora

No. of corpus	Average length of the corpus	Minimum length of the corpus	No. of vocabulary
1868198	382	2	30

#### Pretraining the ELECTRA models

Our method exploits two separate ELECTRA models to separately encode the SMILESs and amino acid sequences into fixed dimensional vectors. In an effort to avoid confusion and awkward phrasing, we describe the procedure of training the ELECTRA model for the SMILESs. The procedure for the pre-trained ELECTRA model for the amino acids sequences is similar.

**Fig. 2:**
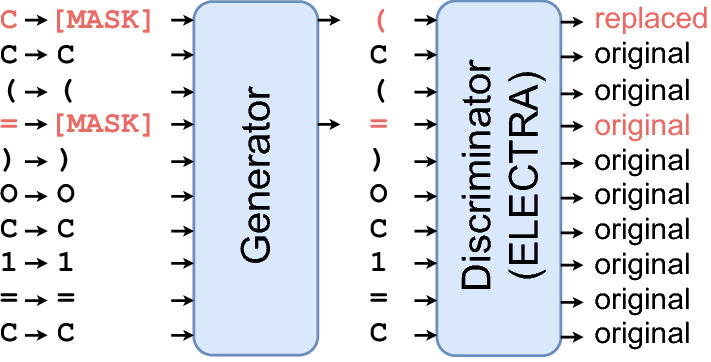
The principle of ELECTRA pre-training

ELECTRA exploits two Transformer encoders as the base structure: one acts as a generator network, and the other acts as a discriminator network. The generator is typically a small, masked language model that produces an output distribution over the tokens. The tokens from a SMILES string are first masked, and some are replaced with a mask symbol [MASK] with a constant probability. Then, the masked tokens are fed into the generator for joint pre-training with the discriminator. The generator network first learns from the masked tokens and then fills the missing tokens with predicted values, but the predicted value may not be the same as the original value. However, the jointly trained discriminator network learns to resolve whether each token is the same as the original one. It uses another Transformer encoder to extract the contextual information as embedding representations, which are used to determine the probability of replacing the tokens, as shown in Fig. 2. After pre-training with SMILES strings, we obtain an ELECTRA-M model as an encoder that can encode each SMILES into a feature vector in downstream tasks. Similarly, we obtain an ELECTRA-P model pre-trained with protein sequences that also encode each protein sequence into a feature vector.

### Input representation

Protein sequence and compound SMILES are fed into the entire framework as input; because the SMILES strings and protein sequences are of different lengths, we truncate them to fixed lengths for effective representation. As in [3], we also choose fixed lengths of 100 for SMILES and 1000 for protein sequences for the benchmark of datasets. We chose these maximum lengths based on the distributions of the datasets so that the maximum lengths cover at least 80% of the proteins and compounds in the datasets. Longer sequences are truncated to these lengths, while shorter sequences are padded with zeros to the fixed lengths.

### Compound SMILES and protein sequence embedding

**Fig. 3:**
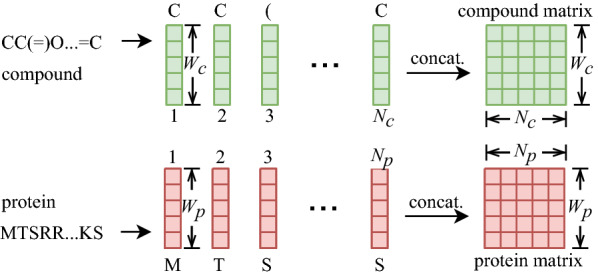
Compound SMILES and protein sequence embedding

With the trained ELECTRA-M and ELECTRA-P models, the input compound SMILES strings and protein sequences are embedded into tensors separately. For individual compounds, the sequence of tokens from their SMILES strings, which represents atoms or structure indicators, is fed into the trained ELECTRA-M model to yield a compound encoding. Specifically, each token, which is one character, is converted to a vector of length $W_c$ by ELECTRA-M, and then a sequence of $N_c$ tokens is converted to a sequence of $N_c$ vectors that are finally concatenated into a $W_c \times N_c$ tensor as the compound representation. In the same way, one protein residue token is encoded into a vector of length $W_p$ by ELECTRA-P, and a protein sequence of length $N_p$ vectors that are concatenated into a $W_p \times N_p$ tensor, as shown in Fig. 3.

### ELECTRA-DTA framework

**Fig. 4:**
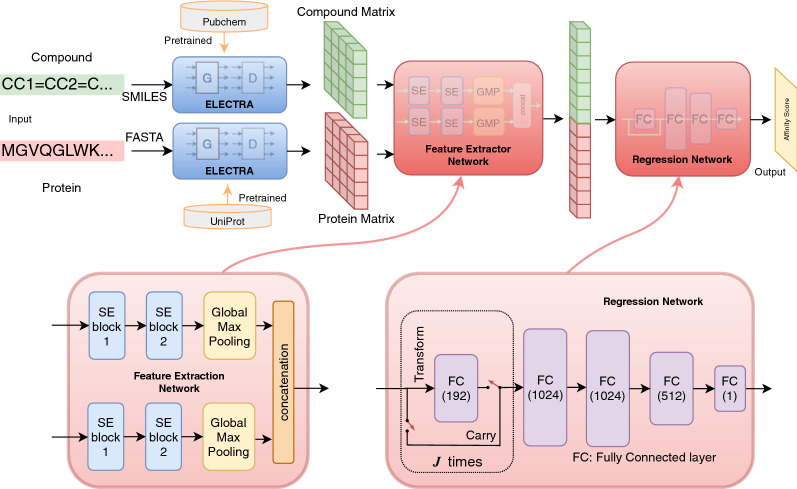
Architecture of the ELECTRA-DTA model

In this work, the ELECTRA-DTA model incorporates two identical feature extractor networks and one regression network, as shown in Fig. 4. Each feature extractor network is a typical convolutional neural network equipped with two layers of stacked squeeze-and-excitation (SE) blocks in addition to one global max pooling filter layer. The feature extractor network takes the compound or protein tensor as input to learn latent features during the supervised training process and then produces feature vectors as a representation. Two feature vectors are then concatenated into a single vector, which is fed into the regression network for the prediction.

**Fig. 5:**
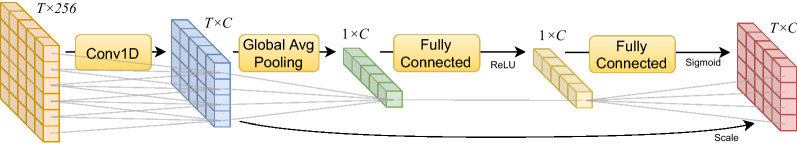
Structure of the squeeze-and-excitation block

Inside the feature extractor network, the main blocks are two SE block layers. These are recently proposed CNN units that can be stacked for extremely effective generalization across different datasets, especially image processing. The SE block improves the joint encoding of image spatial and channel information by removing the spatial dependency with global average pooling to learn a channel-specific descriptor. This block is capable of feature recalibration by using global information to selectively emphasize informative features over the others. The SE block of the ELECTRA-DTA model is shown in Fig. 5. We use one-dimensional convolution to project the input compound or protein tensor $X \in {\mathbb {R}}^{T \times 256}$ into the feature maps $U \in {\mathbb {R}}^{T \times C}$ to perform feature recalibration. This one-dimensional convolution is in the feature direction. Therefore, these feature maps *U* are first squeezed and produce a channel (or feature) descriptor by global average pooling in the feature direction. The interaction information is accumulated in this descriptor. The squeezing operation is followed by an excitation operation with a fully connected layer and ReLU activation, which produces modulation weights from the features by a simple self-gating mechanism. The output of the SE block is in the form of the feature maps *U* scaled by these modulation weights. The stacked SE block further enlarges this interaction information between features.

The regression network contains two tandem parts, as shown in Fig. 4. The first tandem part consists of a stack of fully connected layers with a simple gating mechanism. The second tandem part has four fully connected layers of different sizes. The simple gating mechanism in the first part is adopted from [13] to better regulate information flow in consecutive training epochs. Each layer consists of two gates called a Carry and a Transform that separate information flow to two streams-one modified, the other untouched-to the next iteration. Finally, all information flows through four fully connected layers and then produces the predicted affinity score.

## Model validation

### Datasets

This study evaluated ELECTRA-DTA using three common benchmark datasets: the KIBA dataset [14], the Davis dataset [15], and the BindingDB dataset [16]. The Davis dataset consists of 442 proteins and 68 compounds forming 30,056 DT pairs, while the KIBA dataset contains 229 proteins and 2111 compounds forming 118,254 DT pairs. BindingDB is a web-accessible public dataset, and the filtered version is exploited in this study to maintain consistency with a previous study [4]. Table 3 provides the statistics of these datasets.

**Table 3:** The detailed statistics of the datasets, containing the number of proteins, compounds, interactions and average number of interactions per protein and per compound

	No. Proteins	No. Compounds	No. Interaction	No. interactions per protein	No. interactions per compound	No. inactive interactions	No. non-inactive interactions
Davis	442	68	30056	68	442	2502	27554
KIBA	229	2111	118254	516	56	24828	93426
BindingDB	1620	87461	144525	89	2	47608	96917

However, the versions of these datasets used here share one crucial problem: the same DT sequence has different or duplicate affinity values. Duplicated samples may harm the effectiveness of the deep learning DTA model, while sample inputs (DT pairs) with different labels (binding affinity values) may be detrimental to training the deep learning DTA model. We thus removed these samples from the original datasets. Table 4 describes the details of the refined datasets.

**Table 4:** The detailed statistics of the refined datasets, containing the number of proteins, compounds, interactions and average number of interactions per protein and per compound

	No. Proteins	No. Compounds	No. interaction	No. interactions per protein	No. interactions per compound	No. inactive interactions	No. non-inactive interactions
Davis	361	68	24548	68	61	1649	22899
KIBA	229	2052	117184	511.720	57.107	24543	92641
BindingDB	1615	129109	144525	79.944	1.541	41487	87622

It can be observed that these datasets differ significantly in terms of the number of interactions per protein or compound. For the Davis and KIBA datasets, the average number of interactions per protein and compound is higher than 50, whereas BindingDB contains much fewer interactions for both compounds and proteins, suggesting that the connections between compounds and proteins are sparse, which would make the training of the prediction models challenging. In addition, all of these datasets have skewed distributions.

### Evaluation metrics

The performance of all models was measured by the concordance index [17] (CI), the mean squared error (MSE), Pearson correlation coefficient, the $r_m^2$ index [18, 19] and the area under precision recall (AUPR) curve score. CI indicates the ranking performance of the models and can be calculated by equations (1).

1 $$\begin{aligned} \text {CI}=\frac{1}{Z} \sum _{\delta _{x} > \delta _{y}} h\left( b_{x}-b_{y}\right) , \quad \text{ where } h(m)=\left\{ \begin{array}{ll}1, & \text{ if } \, m > 0 \\ 0.5, & \text{ if }\, m=0 \\ 0, & \text{ if } \, m < 0\end{array}\right. \end{aligned}$$

The $r_m^2$ index defines the possibility of an acceptable model and is calculated by equation (2), where $r_0^2$ and $r^2$ are the squared correlation coefficients with and without intercepts, respectively.

2 $$\begin{aligned} r_{m}^{2}=r^{2} *\left( 1-\sqrt{r^{2}-r_{0}^{2}}\right) \end{aligned}$$

Generally, a model with an $r_m^2$ index greater than 0.5 on a test set can be considered acceptable. The AUPR curve score is generally adopted for binary prediction; therein, we converted the regressions on these datasets into their binary forms by thresholding similarly to DeepDTA. The Pearson correlation (R) is a metric that measures the correlation between two continuous variables.

There are three variant ELECTRA models: ELECTRA small, with 14M parameters, ELECTRA base, with 110M parameters, and ELECTRA large, with 335M parameters. Due to limited computing resources, we only pretrain two ELECTRA small models, one for compounds and another for proteins. We use code from simpletransformers to train these two ELECTRA small models. Unlike with the NLP ELECTRA models, we use the atom tokenization method for the compound SMILESs and protein amino acid sequences. The pre-training task of the ELECTRA small models takes 6 days with a batch size of 256, a learning rate of 1e-5, and an epoch of 100 by using 8 NVIDIA GeForce RTX 2080 Ti GPUs. The choice of hyper-parameters for pretrained ELECTRA models are follows the original paper [7].

The hyper-parameters optimization for the feature extraction block is done for the number of the filters (same for proteins and drugs) searched over [16, 32, 64, 128, 256,512], and the learning rate search over [3e-3,1e-3,5e-4,3e-4,1e-4,1e-5]. The feature extraction block comprises 2 convolutional layers, one with 256 and the other with 512 channels. All convolutional layers apply 3 convolution kernels and are activated through rectified linear unit (ReLU) activation function. The regression block has 4 FC layers with 1024, 1024, 512, and 1 node. Each of the first two FC layers is followed by a dropout layer with a rate of 0.4. These 4 FC layers also use ReLU as the activation function. The Adam optimizer was used to train the parameters of the feature extraction block and regression block with the default learning rate of 0.001 for each DTA dataset. The detailed settings are summarized in Table 5.

**Table 5:** The detailed training settings of ELECTRA-DTA

Parameter	Setting	Parameter	Setting
CNN kernel size	3	Learning rate (lr)	3e-4
Length of SMILES sequence	100	Length of protein sequence	1000
Vector dimension	256	Number of filters	256;512
Epoch	100	Batchsize	256
hidden neurons	1024; 1024; 512	dropout	0.4

### Results

#### Results of predictive performance of random splitting settings

Four baseline approaches were selected for comparison with the proposed ELECTRA-DTA, including two classical machine learning-based prediction models, KronRLS and SimBoost, and two state-of-the-art deep learning-based models, DeepDTA and DeepCDA [20], when using the original datasets. It should be noted that to avoid potential errors from the implementation and training of these models, the results of the baseline approaches reported in the following tables are taken directly from their original publications. As the authors for DeepCDA did not provide details on the hyperparameters of the network, we were unable to reproduce their methods. Therefore, we compared our method with another state-of-the-art method: AttentionDTA [21].Given the output of ELECTRA layer $\text {layer}_k$, we used average of all token vectors from 12 Transformer layers for the feature extractor network.

To quantify the prediction performance in an unbiased and consistent manner, we carried out 5-fold cross-validation similar to that used for DeepDTA, DeepCDA and SimBoost; that is, we split the data arbitrarily into six equivalent parts in which one part is selected as the independent test set. Any one of the remaining parts is used as the validation set and the others as the training set. The purpose of this division is to determine the hyper-parameters via 5-fold cross validation. Tables 6,  7 and  8 report the results of all the evaluated models and their average CI, MSE, R, $r_m^2$ and AUPR curve scores with the original and refined KIBA, Davis and BindingDB datasets, respectively.

**Table 6:** Comparison of all baseline approaches and ELECTRA-DTA on the KIBA datasets

Dataset	Model	CI	MSE	R	$r_m^2$	AUPR
Original KIBA Dataset	KronRLS	0.782	0.411	-	0.342	0.635
	SimBoost	0.836	0.222	-	0.629	0.760
	DeepDTA	0.863	0.194	0.848	0.673	0.788
	WideDTA	0.875	0.179	-	-	-
	DeepCDA	**0.889 (0.002)**	0.176	0.855	0.682 (0.008)	**0.812 (0.005)**
	ELECTRA-DTA	0.889 (0.003)	**0.162**	**0.879**	**0.727 (0.004)**	0.795 (0.006)
refined KIBA Dataset	DeepDTA	0.892 (0.026)	0.152	**0.896**	0.766 (0.085)	0.798 (0.063)
	Attention-DTA	0.880 (0.001)	0.158	0.883	0.742 (0.015)	0.795 (0.003)
	ELECTRA-DTA	**0.892 (0.002)**	**0.143**	0.892	**0.780 (0.014)**	**0.805 (0.005)**

Bold values represent the best performance over all competitive methods

**Table 7:** Comparison of all baseline approaches and ELECTRA-DTA on the Davis datasets

Dataset	Model	CI	MSE	R	$r_m^2$	AUPR
Original Davis Dataset	KronRLS	0.871	0.379	–	0.407	0.661
	SimBoost	0.872	0.282	–	0.644	0.709
	DeepDTA	0.876 (0.004)	0.261	0.846	0.630 (0.017)	0.714 (0.010)
	WideDTA	0.886	0.262	–	–	–
	DeepCDA	0.891 (0.003)	0.248	**0.857**	0.649 (0.009)	**0.739 (0.006)**
	ELECTRA-DTA	** 0.897 (0.003)**	**0.238**	0.844	**0.671 (0.032)**	0.698 (0.010)
refined Davis Dataset	DeepDTA	0.882 (0.016)	**0.191**	**0.843**	0.690 (0.035)	**0.695 (0.03)**
	Attention-DTA	0.888 (0.007)	0.195	0.836	**0.697 (0.005)**	0.677 (0.022)
	ELECTRA-DTA	**0.896 (0.002)**	0.195	0.838	0.637 (0.048)	0.685 (0.026)

Bold values represent the best performance over all competitive methods

**Table 8:** Comparison of all baseline approaches and the ELECTRA-DTA on the BindingDB Dataset

Dataset	Model	CI	MSE	R	$r_m^2$	AUPR
Original BindingDB Dataset	DeepDTA	0.812 (0.002)	0.832	0.824	0.623 (0.02)	0.443 (0.01)
	DeepCDA	0.822 (0.001)	0.844	0.808	0.631 (0.002)	0.459 (0.003)
	ELECTRA-DTA	**0.832 (0.004)**	**0.693**	**0.852**	**0.645 (0.012)**	**0.807 (0.002)**
refined BindingDB Dataset	DeepDTA	0.826 (0.001)	0.703	0.845	0.669 (0.004)	0.795 (0.003)
	Attention-DTA	0.804 (0.003)	0.844	0.811	0.619 (0.009)	0.764 (0.004)
	ELECTRA-DTA	**0.837 (0.002)**	**0.650**	**0.860**	**0.670 (0.027)**	**0.811 (0.001)**

Bold values represent the best performance over all competitive methods

For the original KIBA dataset, the proposed ELECTRA-DTA and DeepCDA achieve the best performance metrics, with ELECTRA-DTA outperforming DeepCDA by 0.014, 0.024 and 0.045 for MSE, R, and the $r_m^2$, respectively, while DeepCDA achieved the best CI and AUPR curve score. Both models achieved much better prediction performance than DeepDTA, SimBoost and KronRLS. As seen from Table 6, the proposed ELECTRA-DTA achieved values of 0.892, 0.143, 0.892, 0.780, and 0.805 for CI, MSE, R, $r_m^2$ and AUPR curve score, respectively, for the refined KIBA dataset. Except for the R metric, our proposed method outperformed DeepDTA and AttentionDTA.

For the original Davis dataset as in Table  7, ELECTRA-DTA outperformed the next-best model in terms of the CI and $r_m^2$ index by 0.006 and 0.035, respectively. Additionally, the MSE error was 0.238, which was lower than that of the state-of-the-art methods. It should also be noted that DeepCDA performed better than ELECTRA-DTA in terms of the AUPR curve score. For the refined Davis dataset, the proposed ELECTRA-DTA-AVG model had the highest CI. Our methods did not generate the best results for the other metrics, but the differences were small.

Table 8 summarizes the evaluation metrics of the various methods for the original and refined BindingDB datasets. ELECTRA-DTA outperformed the baseline approaches for all evaluation metrics. For the original dataset, ELECTRA-DTA obtained a 0.01 increase in the CI, while the MSE value of our method was 0.151 lower than that of DeepCDA. The most substantial improvement was observed for the AUPR curve score, which increased from 0.459 for DeepCDA to 0.807. For the refined BindingDB dataset, our ELECTRA-DTA exceeded DeepDTA and AttentionDTA for all evaluation metrics, with ELECTRA-DTA yielding the best performance. Among all evaluation metrics, MSE has the most noticeable difference, which decreasing from 0.844 for AttentionDTA to 0.626. The improvement in the CI was distinct, achieving the highest value (0.837) over the baselines. The $r_m^2$ index, R and the AUPR curve score improved substantially relative to those of AttentionDTA and DeepDTA. It should be noted that ELECTRA-DTA achieved better results in the BindingDB dataset in all metrics than the baseline models, likely due to differences in the dataset distribution. According to an analysis of these datasets, the BindingDB dataset has sparser samples, thereby making it more difficult to train prediction models. This suggests that our model has more robustness and reliability.

Interestingly, we observed that the CIs for DeepDTA and ELECTRA-DTA increased significantly from the original to the refined datasets. The reason for this is that different proteins or drugs have identical representations in the original datasets, and consequently, in the deep learning-based methods, the network becomes confuses as to how to learn an effective representation for these proteins or drugs.

According to the reported results in Tables 6, 7 and 8, ELECTRA-DTA and DeepCDA consistently performed the other DTA models in terms of all metrics, with ELECTRA-DTA-AVG being responsible for 10 of the 15 best scores for the refined datasets, indicating that our model has more reliable and accurate predictive performance than the other models.

#### Ablation Study

The use of pretrained-embeddings in theory lets the model leverage a much larger training set to help the neural network “understand” biochemistry. To understand the contribution of the pretrained-embeddings to the overall performance in our method, we replaced the pretrained-embeddings with one-hot encoding from our model. We called the replaced version as Onehot-DTA in the following. We conducted the ablation experiment using the refined datasets. As shown in Table 9, the model using the pretrained embedding feature has an improvement of 0.046, 0.009 and 0.007 on CI metric for Davis, KIBA and BindingDB datasets. This ablation experiment emphasizes the advantage of using the pretrained embedding as the proteins and drugs representations, which can provide high-level protein information and molecular information.

**Table 9:** Results of ablation experiments

Dataset	method	CI	MSE	R	$r_m^2$	AUPR
Davis	ELECTRA-DTA	**0.896 (0.002)**	**0.195**	**0.838**	**0.637 (0.048)**	**0.685 (0.026)**
	Onehot-DTA	0.850 (0.005)	0.301	0.739	0.525 (0.018)	0.561 (0.029)
KIBA	ELECTRA-DTA	**0.892 (0.002)**	**0.143**	**0.892**	**0.780 (0.014)**	**0.805 (0.005)**
	Onehot-DTA	0.883 (0.002)	0.157	0.881	0.744 (0.012)	0.792 (0.003)
BindingDB	ELECTRA-DTA	**0.837 (0.002)**	**0.650**	**0.860**	**0.670 (0.027)**	**0.811 (0.001)**
	Onehot-DTA	0.830 (0.001)	0.700	0.849	0.659 (0.037)	0.799 (0.004)

Bold values represent the best performance over all competitive methods

#### Results of predictive performance of cold splitting settings

One of the key difficulties in DTA prediction is generalization of the model and the discovery of the binding affinity for unseen drugs or targets. Therefore, in these experiments, three splitting schemes were used for all refined datasets:

1. Cold-drug setting: Every drug in the test set is absent in the training set.
2. Cold-target setting: Every target in the test set is absent from the training set.
3. Blind splitting: the target and the drug are both absent in the training set.

The cold-drug, cold-target and blinding splits deliver realistic and more difficult appraisal schemes for the DTA problem. In the real DTA prediction setting, the data redundancy problem caused by similar proteins or drugs may lead to “easy predictions”, which may mislead the performance evaluation of different algorithms. To conduct an objective evaluation, we use the single-linkage clustering [22] to ensures that the compounds (or proteins) within the same cluster, which share high similarities, are either all used in the training set, or all used in the test set. More specifically, the distance between two protein $p_i$ and $p_j$ is defined as

3 $$\begin{aligned} Distance(p_i,p_j) = 1 - \frac{SW(p_i,p_j)}{\sqrt{SW(p_i,p_i)SW(p_j,p_j)}} \end{aligned}$$

where $SW(\cdot ,\cdot )$ stands for the Smith-Waterman alignmen score [23] between two sequences.

The distance between a pair of compounds $c_i,c_j$ is defined as

4 $$\begin{aligned} Distance(c_i,c_j) = 1 - \text {Jaccard}(MF(c_i), MF(c_j)) \end{aligned}$$

where $MF(\cdot )$ stands for the Morgan fingerprints calculated by RDKit [24] and $\text {Jaccard}(\cdot ,\cdot )$ denotes the Jaccard similarity.

The single-linkage clustering threshold in this experiment is 0.3. Compounds (proteins) belonging to the same cluster were assigned to same folds, so that compounds (proteins) in the test fold would not be similar to those in the training fold. In the cold-drug setting, there can be no compound protein pairs with compounds from the same cluster between the training set, validation set and test set. Similar settings exist in the cold-target setting. For the cold-drug and the cold-target settings, the ratio of training set, validation set and test set is approximately 7:1:2. In the blinding setting, both compound clusters and protein clusters cannot be shared across training, validation and test sets. We chose nine-fold cross-validation for the blinding setting because no protein or compound clusters can be shared between training and test sets in this case. In particular, We divided protein clusters into three folds at random and then divided compound clusters into three folds within each fold of protein clustering. The compound-protein pairs were then partitioned into a $3\times 3$ grid. We chose a single grid as the test set and eliminated the four grids that shared protein or peptide clusters with the test set. Finally, we trained the model using the remaining four grids. There was no shared protein or peptide cluster between the training and test sets.

**Fig. 6:**
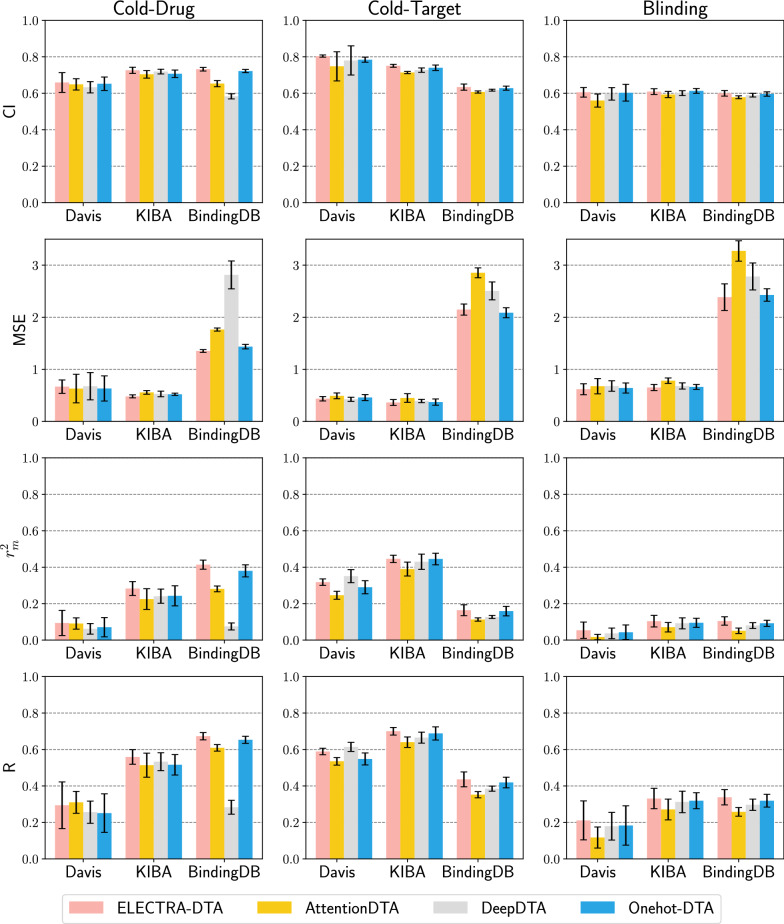
Performance evaluation for ELECTRA-DTA and baseline methods on cold splitting settings, on both Davis, KIBA and BindingDB datasets. The error bar shows the standard error

The quantitative results for ELECTRA-DTA and baseline methods are shown in Fig. 6 (see Additional file 1: Table for more details). Note that the AUPR scores were not comparable among these three settings, due to the different distributions of binding affinity values. For these three cold splitting settings, ELECTRA-DTA attained the best performance in most of our experiments. It can also be seen from the Fig. 6 that ELECTRA-DTA is close to other methods in terms of CI values under the blinding setting, but from the perspective of MSE, $r_m^2$ and R scores, our method still has certain advantages. Compared to the random splitting settings, the performance of all methods is decreased drastically. Besides, all the methods could not obtain good $r_m^2$ in these three cold splitting settings. The main results may lie in that the test set not only contain new proteins or/and compounds, but also consist of very dissimilar proteins or/and compounds compared to the training set. And the machine learning algorithm requires that the training set and test set come from the same distribution. These results were consistent with the previous study [20, 25, 26].

We also noticed that Figure 6 reports a seemingly contradictory phenomenon: all methods perform better in the cold-target setting with the Davis dataset but worse with the BindingDB dataset than in the cold-drug setting. We see that the prediction performance of the deep learning-based methods is correlated to the data distribution of the dataset. Combined with the data in Table 4, it can be seen that when the number of drugs in the training set is large and the number of targets is small, the cold-target splitting scheme tends to yield a more challenging problem than the cold-drug splitting scheme; on the other hand, when there are many targets and few drugs, the cold-target predictions appear to be better than the cold-drug predictions. We hypothesize that the reason behind such contradictory results could be that the models become more stable with greater knowledge on entities (drugs or targets) in the training sets. In addition, we also observe an interesting phenomenon in which the gap between the cold-drug and cold-target predictions is different over the KIBA and Davis datasets. The cold-drug and cold-target prediction CIs are similar in the KIBA dataset, even though it has 2052 drugs and only 229 targets. Along this line, one might conclude that the model needs many more types of drugs than targets to learn their chemical representations.

Additionally, we realized that the blinding split schemes that had fewer drug-target pairs turned out to be the most challenging for the deep learning based models. We can observe that all the models’ performance decreased drastically while our methods usually exhibited a relatively stable performance on the three datasets. Such test results suggested that ELECTRA-DTA can achieve better and more robust performance than the baseline methods under all cross-validation settings.

As shown in Fig. 6, the performance of Onehot-DTA was lower compared with that of ELECTRA-DTA, demonstrating that the pretrained embedding with ELECTRA indeed plays a key role in DTA prediction task. Thus further supporting the the proposed pretrained ELECTRA model can extract the abundant information. In summary, ELECTRA-DTA has the best performance compared with the baselines and variants, which completely signifies that the pretrained embedding is beneficial to DTA prediction tasks.

#### Case study on drug repurposing for COVID-19

**Fig. 7:**
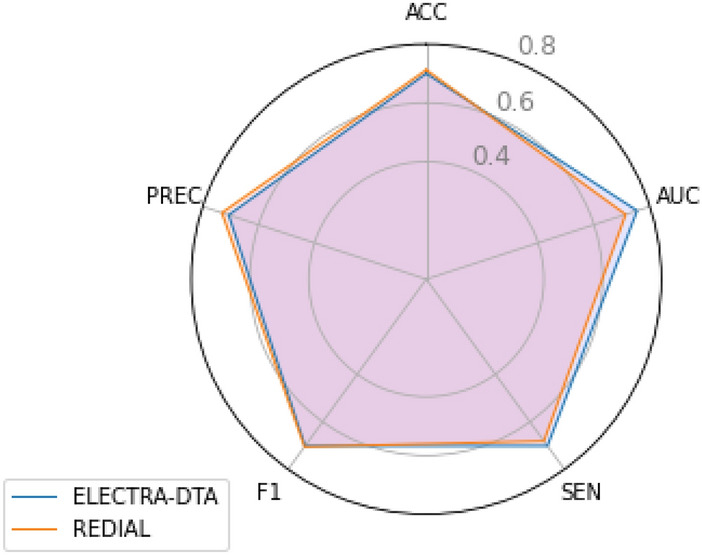
Radar plot of the ELECTRA-DTA and REDIAL models

We conducted a case study to showcase the use of our proposed ELECTRA-DTA for biomedical researchers for the repurposing of existing drugs. Here, we took key protein 3CL from SARS-CoV-2 as our targets and selected a potential drug dataset from the work of REDIAL [27]. It should be noted that the dataset from REDIAL only provides the activate/inactivate for the drug-protein pair, so, we changed our method to a classification model. Specifically, we replaced the loss function of MSE with binary cross entropy, and set the activation function of the last layer as sigmoid. The performance of our method was evaluated by several classical classification evaluation metrics, such as sensitivity (SEN), accuracy,(ACC), F1-score (F1), precision (PREC), and the area under the receiver operating characteristic curve (AUC). The radar plots of ELECTRA-DTA and REDIAL models are shown in Fig. 7. It can be seen from Fig. 7 that the performance of our method is basically similar to that of REDIAL. Our method achieves higher AUC and sensitivity but lower precision. The behind reason can be attributed to the following aspects:

- The REDIAL uses a consensus model based on 15 classifiers and 22 features;
- Our model was originally designed for regression tasks, which pays more attention to the ranking between data. So we get a higher AUC. A higher AUC means the model can order the data very well.

To confirm the utility of our models, we also used the additional dataset of 3CL (Mpro) inhibitors: ebselen, disulfiram, tideglusib, carmofur, shikonin and PX-12. Among these six inhibitors, our ELECTRA-DTA correctly predicted disulfiram, tideglusib, shikonin and PX-12 as actives.

## Discussion

This study presented ELECTRA-DTA, a DTA prediction approach with two trained ELECTRA models for encoding protein amino acid sequences and compound SMILESs separately in an unsupervised manner. For compound SMILES strings, all characters found in different SMILESs were treated as the vocabulary, and selected compounds from the PubChem dataset were used as a corpus to obtain vector representations of each character, subsequently yielding a vector sequence for each compound SMILES. The unsupervised training on a large-scale sample space incorporated additional contextual information, including potential substructures or function groups. The ELECTRA-based embedding mechanism and the design of our network architecture ensured a better predictive ability than that of state-of-the-art baseline methods; moreover, they provide a feasible encoding module that can be stacked over other feature representation networks as the embedding layer for protein sequences or compound SMILESs in various learning tasks. In addition, they can also effectively train a new embedding model by constructing domain-specific corpora for specific downstream tasks.

## Supplementary Information

**Additional file 1: Table S1**. Performance comparison of ELECTRA-DTA with the baselines through in the cold splitting settings.

## Data Availability

Source code and datasets are available at Github.
