# Supplementary material for: ELECTRA-DTA: a new compound-protein binding affinity prediction model based on the contextualized sequence encoding
Source: J Cheminform. 2022 Mar 15;14:14. doi: 10.1186/s13321-022-00591-x (PMC8922401; doi:10.1186/s13321-022-00591-x)
Supplement: Supplementary file 1 — Additional file 1: Table S1. Performance comparison of ELECTRA-DTA with the baselines through in the cold splitting settings. [file 13321_2022_591_MOESM1_ESM.pdf]

Supplementary Table for "ELECTRA-DTA: A new compound-protein binding affinity prediction model based on the contextualized sequence encoding"

Table 1: Performance comparison of ELECTRA-DTA with the baselines through in the cold splitting settings.

Dataset	Setting	Method	CI	MSE	r_m^2	R
Davis	Cold-Drug	ELECTRA-DTA	**0.659(0.055)**	0.667(0.129)	**0.094(0.069)**	0.294(0.128)
		Onehot-DTA	0.653(0.037)	0.632(0.241)	0.071(0.053)	0.251(0.106)
		DeepDTA	0.633(0.030)	0.675(0.262)	0.062(0.029)	0.256(0.061)
		AttentionDTA	0.649(0.031)	**0.630(0.274)**	0.091(0.031)	**0.310(0.060)**
	Cold-Target	ELECTRA-DTA	**0.804(0.006)**	0.435(0.042)	0.318(0.018)	0.589(0.018)
		Onehot-DTA	0.784(0.014)	0.458(0.058)	0.290(0.036)	0.548(0.033)
		DeepDTA	0.780(0.008)	**0.424(0.039)**	**0.351(0.036)**	**0.614(0.025)**
		AttentionDTA	0.748(0.008)	0.490(0.056)	0.246(0.022)	0.535(0.021)
	Blinding	ELECTRA-DTA	**0.605(0.026)**	**0.617(0.105)**	**0.054(0.045)**	**0.211(0.107)**
		Onehot-DTA	0.603(0.046)	0.641(0.097)	0.043(0.040)	0.183(0.108)
		DeepDTA	0.597(0.034)	0.679(0.101)	0.0371(0.029)	0.179(0.076)
		AttentionDTA	0.560(0.036)	0.676(0.147)	0.017(0.0148)	0.117(0.058)
KIBA	Cold-Drug	ELECTRA-DTA	**0.727(0.018)**	**0.480(0.029)**	**0.283(0.038)**	**0.559(0.04)**
		Onehot-DTA	0.707(0.021)	0.520(0.022)	0.243(0.055)	0.516(0.056)
		DeepDTA	0.719(0.013)	0.527(0.054)	0.241(0.039)	0.533(0.049)
		AttentionDTA	0.704(0.021)	0.556(0.036)	0.225(0.057)	0.514(0.066)
	Cold-Target	ELECTRA-DTA	**0.751(0.008)**	**0.363(0.057)**	**0.446(0.020)**	**0.700(0.021)**
		Onehot-DTA	0.740(0.015)	0.370(0.061)	0.445(0.032)	0.688(0.036)
		DeepDTA	0.727(0.012)	0.391(0.032)	0.430(0.042)	0.665(0.030)
		AttentionDTA	0.714(0.006)	0.449(0.083)	0.390(0.038)	0.640(0.029)
	Blinding	ELECTRA-DTA	0.609(0.016)	**0.650(0.059)**	**0.104(0.032)**	**0.331(0.056)**
		Onehot-DTA	**0.613(0.013)**	0.661(0.048)	0.095(0.025)	0.319(0.044)
		DeepDTA	0.601(0.019)	0.680(0.059)	0.092(0.030)	0.312(0.059)
		AttentionDTA	0.593(0.017)	0.781(0.053)	0.071(0.026)	0.271(0.057)
BindingDB	Cold-Drug	ELECTRA-DTA	**0.732(0.010)**	**1.352(0.027)**	**0.414(0.025)**	**0.673(0.020)**
		Onehot-DTA	0.723(0.009)	1.435(0.043)	0.380(0.033)	0.653(0.0194)
		DeepDTA	0.583(0.014)	2.812(0.268)	0.075(0.019)	0.283(0.038)
		AttentionDTA	0.702(0.008)	1.763(0.029)	0.281(0.016)	0.609(0.018)
	Cold-Target	ELECTRA-DTA	**0.634(0.017)**	2.147(0.107)	**0.164(0.030)**	**0.436(0.041)**
		Onehot-DTA	0.628(0.011)	**2.086(0.096)**	0.159(0.026)	0.419(0.029)
		DeepDTA	0.617(0.005)	2.505(0.172)	0.127(0.008)	0.385(0.014)
		AttentionDTA	0.607(0.006)	2.853(0.095)	0.113(0.009)	0.352(0.017)
	Blinding	ELECTRA-DTA	**0.600(0.015)**	**2.384(0.256)**	**0.105(0.023)**	**0.338(0.042)**
		Onehot-DTA	0.596(0.012)	2.426(0.120)	0.092(0.017)	0.319(0.035)
		DeepDTA	0.590(0.010)	2.782(0.259)	0.080(0.016)	0.297(0.031)
		AttentionDTA	0.578(0.008)	3.272(0.195)	0.051(0.015)	0.258(0.024)
